# Acute intestinal intussusception among children under five years of age admitted in an Ouagadougou hospital, Burkina Faso, 2008-2013: epidemiological, clinical and therapeutic aspects

**DOI:** 10.11604/pamj.supp.2021.39.1.25270

**Published:** 2021-07-28

**Authors:** Tapsoba Wendlamita Toussaint, Albert Wandaogo, Idrissa Clétus Yaméogo W, Isso Ouédraogo, Somkièta Modeste Francis Ouédraogo, Olivier Zampou, Bernadette Béré, Negar Aliabadi, Eyal Leshem, Moumouni Nikièma, Ma Ouattara, Jason M Mwenda, Isidore Bonkoungou, Emile Bandré, Umesh D Parashar, Jacqueline E Tate

**Affiliations:** 1 Centre Hospitalier Universitaire Pédiatrique Charles de Gaulle, Ouadougou, Burkina Faso,; 2 Centers for Disease Control and Prevention, Atlanta, United States,; 3 Ministry of Health, Burkina Faso,; 4 World Health Organization, Burkina Faso,; 5 World Health Organization, Regional Office for Africa, Brazzaville, Congo,; 6 National Public Health Laboratory, Burkina Faso

**Keywords:** Intestinal intussusception, Burkina Faso, child, incidence, mortality

## Abstract

**Introduction:**

acute intestinal intussusception is a life-threatening surgical condition. In some settings, rotavirus vaccines have been associated with a low-level increased risk of intussusception. We describe the epidemiology, clinical manifestations and management of intussusception in a tertiary referral hospital in Burkina Faso prior to the introduction of rotavirus vaccine in October 2013.

**Methods:**

we retrospectively reviewed medical records of all children under 5 years of age treated at the Charles de Gaulle Pediatric Hospital for intussusception meeting the Brighton level 1 diagnostic criteria, from October 31st, 2008 to October 30th, 2013. We report the incidence of intussusception as well as descriptive characteristics of these cases.

**Results:**

a total of 107 Brighton level 1 intussusception cases were identified, representing a hospital incidence of 21.4 cases / year. There were 69 males and 38 females (sex ratio of 1.8), with a median age of 8 months (range 2 months to 4 years). Sixty-two percent of intussusception cases occurred among infants (n = 67 cases). The average time from symptom onset to seeking medical consultation was 3.8 days +/- 2.7 (range 0 to 14 days). Treatment was mainly surgical (105 patients, 98.1%) with 35 patients (32.7%) undergoing intestinal resection. Thirty-seven patients (35.5%) experienced post-operative complications. The mortality rate was 9.3%. Intestinal resection was a risk factor for death from intussusception.

**Conclusion:**

in this review of intussusception hospitalizations prior to rotavirus vaccine introduction in Burkina Faso, delays in seeking care were common and were associated with mortality.

## Introduction

Acute intestinal intussusception is the leading cause of acute intestinal obstruction in infants and young children [1-4]. An expedient diagnosis is of paramount importance as the classic clinical triad described by Ombredanne- paroxysmal abdominal pain, vomiting and rectorrhagia- reflects an already advanced stage of illness [5]. Intussusception prognosis is relatively better in developed countries where diagnosis is early and treatment is non-operative [2]. However, in developing countries, intussusception cases have a mortality rate of up to 25% [3]. The etiology is unknown in more than 90% of cases [6]. Rotavirus vaccines have been associated with a low-level increased risk of intussusception in post-marketing studies in some settings that have introduced these vaccines in their national immunization programs. The first commercial rotavirus vaccine, used only in the United States (RRV-TV, RotaShield, Wyeth Laboratories, PA), was withdrawn from the market in 1999 due to an increased risk of IS of 1 case per 10,000 vaccinated children [7, 8]. Following the introduction of the pentavalent (RV5, RotaTeq, Merck and Co, Inc, Pennsylvania, PA) and monovalent (RV1, Rotarix, GSK Biologicals, Rixensart, Belgium) rotavirus vaccines, a smaller risk of 1 to 2 excess cases per 100,000 children vaccinated was reported in some settings, including the United States, Australia and Latin America [9-11]. No such association was found with Rotarix use in a recent multi-country post-licensure evaluation in sub-Saharan Africa [12].

Burkina Faso started introduction of RV5 into its Expanded Program on Immunization from 31 October 2013. Although the reported risk for intussusception is considered very low with the currently available rotavirus vaccines, the World Health Organization (WHO) has recommended that baseline epidemiological studies be conducted as a prelude to prospective intussusception surveillance instituted with introduction of rotavirus vaccines into national immunization programs. In this study we describe the epidemiology, clinical features, and management of intussusception cases at a large pediatric tertiary referral hospital in Ouagadougou, Burkina Faso. We also evaluated potential risk factors for mortality among intussusception cases and evaluate intussusception treatment in this hospital to identify factors to target for improvements in management.

## Methods

This study was conducted at the Charles de Gaulle Pediatric University Hospital in Ouagadougou (CDH - CHU). CDG-CHU is a 134 bed tertiary pediatric referral hospital which was the only specialized pediatric surgery center with 24 beds in Burkina Faso during the study period. Intussusception cases were retrospectively identified from October 31, 2008 to October 30, 2013, the five years preceding the introduction of rotavirus vaccine in the national immunization program. We abstracted data from the medical records of all children under 5 years of age hospitalized for intussusception as defined by the Brighton Level 1 criteria for diagnostic certainty, namely: a demonstration of the intussusception during the surgical procedure, or an enema demonstration or an ultrasound demonstration with typical images before and after reduction, or confirmation of the intussusception at autopsy (pathological criteria) [2, 6]. Cases of recurrent intussusception and cases with incomplete medical records were excluded.

Despite the retrospective nature of the study, case registration was exhaustive. A team of three surgical residents identified intussusception cases by comparing information from the surgical emergency registers, the outpatient clinic, the hospital unit and the operating room register. The monthly mortality reports for the pediatric surgery service were also reviewed. The cases identified in these registers who were lost to follow-up were recalled for a clinical evaluation. Data collected included demographic, clinical, diagnostic, and treatment characteristics. Bowel resection was defined as a limited resection when the intestinal segment removed was less than or equal to 30 cm and defined as an extended resection when the segment was greater than 30 cm.

The data were entered and processed in Excel and Epi-Info version 7.1.4.0. Descriptive statistics are presented. We also evaluated whether treatment delay times and intestinal resection were risk factors for death. Chi-square tests were used to assess the statistical significance in these comparisons. P-values < 0.05 were considered statistically significant. This study was approved by the ethical review committee of the Ministry of Health in Ouagadougou. This evaluation was determined to be public health non-research during CDC human subjects review.

**Disclaimer:** the findings and conclusions in this report are those of the authors and do not necessarily represent the official position of the Centers for Disease Control and Prevention or World Health Organization (WHO).

## Results

**Figure 1:**
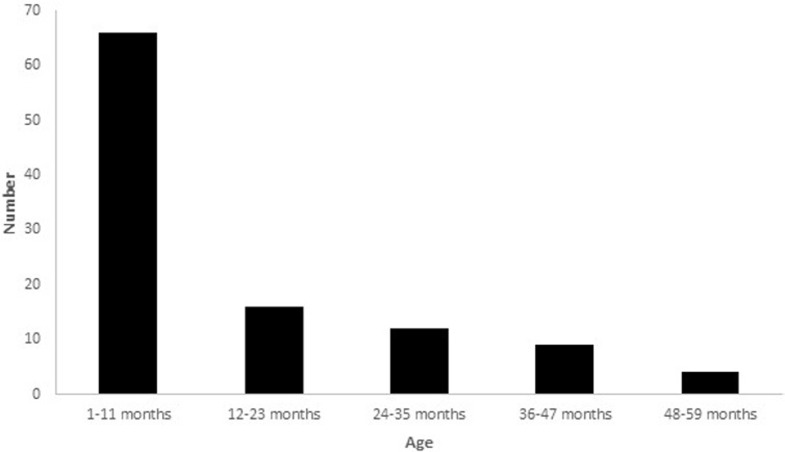
age distribution of children hospitalized with acute intestinal intussusception (n=107)

**Figure 2:**
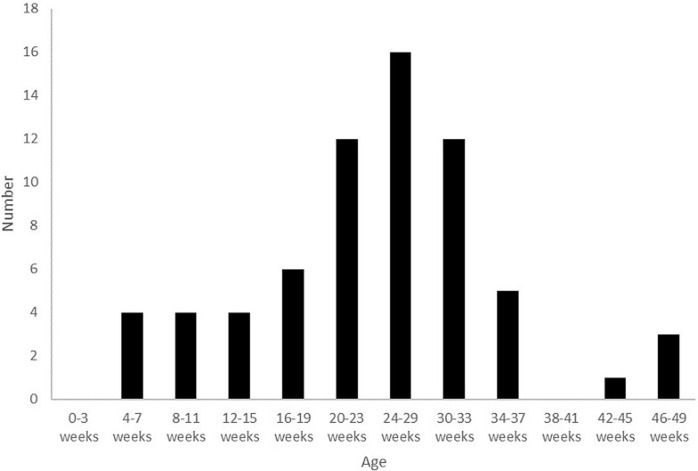
age distribution of infants hospitalized with acute intestinal intussusception (n=67)

We recorded a total of 107 Brighton level I cases of acute intestinal intussusception in 5 years, with an average hospital incidence of 21.4 cases / year. There were 69 boys and 38 girls (sex ratio of 1.8), with an average age of 14.4 months +/- 12.6 (range 2 months to 4 years). The median age was 8 months (Figure 1). The majority of IS cases occurred among infants, with 67 cases (62.6%) diagnosed in this age group, representing a hospital incidence of 13.4 cases / year. The average age for infants diagnosed with IS was 6.5 months +/- 2.28. No IS cases were identified in children < 2 months of age (Figure 2).

**Figure 3:**
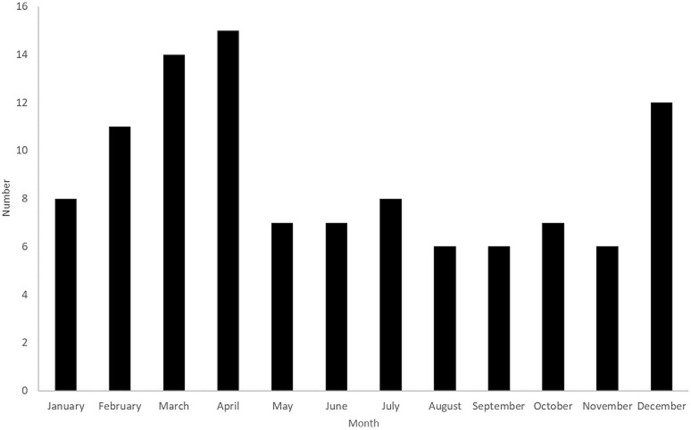
monthly distribution of acute intestinal intussusception cases (n=107)

During the 8-month dry season (October to May) 80 cases (75%) occurred compared with 27 cases (25%) during the 4-month wet season (from June to September) (Figure 3). The time between symptom onset and seeking medical care, specified for 105 patients (98.1%), was on average 3.8 days +/- 2.7 (range 0 days to 14 days). The classic Ombredane triad (paroxysmal abdominal pain, vomiting and rectorrhagia) was present in 26 patients (24.3%) (Table 1). A history of recent acute upper respiratory infection (URI) was noted in three patients (2.8%). In the other 104 patients, the presence or absence of recent URI was not specified in the medical file. All patients received antibiotic treatment. One patient (0.9%) had a spontaneous resolution of the IS before any reduction was attempted. In this patient, an ultrasound confirmed the diagnosis of ileo-ileal intussusception with characteristic images on a cross section and sandwiched on a longitudinal section. While awaiting surgery, this patient improved clinically and a follow-up ultrasound confirmed the spontaneous reduction. A hydrostatic enema with tap water under ultrasound guidance was performed in two patients (1.9%), one of which was successful. The successful case was in a 20-month-old infant. For the failed case, a 7-month-old infant, the definitive treatment was surgical.

A coeliotomy was performed in 105 patients (98.1%) (Table 2). Intraoperative exploration found complete spontaneous reduction in 12 patients with stigmata of parietal ischemia-type invagination, circumferential narrowing groove or simple localized redness. The latter had signs of intussusception on preoperative ultrasound. The intestine was viable in 75% of patients and these patients had the shortest time from symptom onset to consultation (Table 3). Intestinal resection was performed in 35 patients (32.7%). It was extended in 15 (14%) patients and limited in 20 (18.7%). Among the extensive ileocolic resections, two extended to the transverse colon. No pathological findings were found. Bowel resection was followed by an immediate anastomosis in 24 cases and an ileostomy in 11 cases with secondary restoration of digestive continuity. Organic causes were identified in 4 patients (3.7%). Two patients, a 6 months old girl and a 7 years old boy presented tumoral shaped intussusception at the terminal part of the ilium. The third patient, who was a 7 months old boy had a Meckel´s diverticulum. The last one, a 3 years old girl, presented a colic diverticulum. With an average follow-up time of 1.4 months +/- 1.2 (range 15 days - 7 months), 54 (50.5%) patients had an uncomplicated post-operative course. Thirty-seven patients (35.5%) had complications, including post-operative infection, intestinal occlusion, and septicemia (Table 4). Recurrence was observed in 5 patients (4.7%). These last required a surgical revision, which was successful.

**Table 1:** summary of clinical findings of acute intestinal intussusception cases (N=107)

		Number	Percentage (%)
**Symptoms**	abdominal pain	89	83
	rectorrhagia	58	54.2
	vomiting	57	53.3
	diarrhea	33	30.8
**physical examination findings**	deterioration of the general status	25	23.4
	coma	2	2.8
	Moderate to severe dehydration	46	43
	anemia	57	53.3
	fever	54	52
	abdominal distention with resistance	46	43
	soft abdomen	58	54.2
	abdominal guarding	3	2.8
	Externalization of intussusceptum to the anus	4	3.8
	intussusceptum perceived on palpation of the abdomen	47	43.9
	intussusceptum perceived by digital rectal examination	12	11.2
	intussusceptum not perceived	44	41.1
	blood detected on rectal examination	62	57.9
**Biological examination findings**	anemia	92	86
	leucocytosis	85	79.4
	chemistry panels performed	54	50.5
	hypocalcemia	2	1.8
	hypokalemia	1	0.9
	hyponatremia associated with hypochloremia	1	0.9
**Ultrasonographical and radiographical findings**	Confirmation of intussusception by ultrasonography	81	75.5
	air-fluid levels on abdominal radiograph	43	40.2

**Table 2:** surgical details and procedures of acute intestinal intussusception cases (N=105)

		Number	Percentage (%)
**Types of IS**	colo-colic	11	10.5
	ileo-caecolic	68	64.8
	ileo-colic	5	4.7
	ileo-ileal	9	8.6
	spontaneous reduction	12	11.4
**Status of the intestine**	viable	80	76.2
	viable with œdema	2	1,9
	necrotic	12	11,4
	perforated	11	10.5
**Other operative findings**	Meckel´s Diverticulum	1	0.9
	terminal Ilium in tumor shaped	2	1.9
	colic diverticulum	1	0.9
	full common mesentery	1	0.9
	failure of colonic attachment	4	3.8
	mobile caecum	1	0.9
	mesenteric lymphadenopathy	32	30.5
**Procedures**	Manual desinvagination	23	21.9
	Manual desinvagination + appendicectomy	35	33.3
	Intestinal resection + anastomosis	24	22.9
	Intestinal resection + ileostomy	11	10.5

**Table 3:** acute intussusception cases distribution from status of intestine and time of consultation (N=107)

		Number	Percentage (%)	Time from symptom onset to consultation (day)
**Status of the intestine**	viable	80	74.7	3.6
	viable with œdema	2	1.9	8
	necrotic	12	11.2	4.5
	perforated	11	10.3	4.5
	not explored	2	1.9	1
**Total**		107	100	3.8

N = 107 Not explored = 2 patients were not treated by surgery.

**Table 4:** postoperative complications of acute intestinal intussusception (n=105)

	Number	Percentage (%)
Septicemia	3	2.9
Severe undernutrition	1	0.9
Surgical site infection	20	19.1
Postoperative peritonitis	4	3.8
Evisceration	1	0.9
Eventration	2	1.9
Bowel obstruction	6	5.7

Ten deaths were observed, resulting in a mortality rate of 9.3%. The causes of death included postoperative complications, specifically sepsis, decompensated anemia and metabolic disorders. The average postoperative delay for these deaths was 5 days (range 1 to 16 days). The average age of deceased children was 12.2 months (range 2 months to 48 months). We evaluated the time between symptom onset and medical consultation as well as age and intestinal resection as risk factors for mortality among the deceased and living cases (Table 5). Intestinal resection increased the risk of mortality. Mortality also increased with increased time until presentation but this trend was not statistically significant.

**Table 5:** risk factors for mortality among cases of acute intestinal intussusception

			Deceased (N=10)	Living (N=97)	P-value
**Risk factors**	Age	1 - 11 months	7 (70%)	60 (61.9%)	0.42
		12 - 23 months	2 (20%)	19 (19.5%)	
		24 - 35 months	0	15 (15.5%)	
		36 - 48 months	1 (10%)	3 (3.1%)	
	Time from symptom onset to consultation	0 - 23 h	0	18 (18,6%)	0.06
		24 h - 47 h	2 (20%)	36 (37.1%)	
		> 48 h	8 (80%)	43 (44.3%)	
	Intestinal resection		6 (60%)	28 (29%)	0.04

## Discussion

We observed a hospital incidence of intussusception (21.4 cases / year). In an earlier retrospective study from our surgical hospital in Burkina Faso, a lower incidence of 16.2 cases/year was observed [13]. This difference could be due to differences in the rigor of case finding in retrospective studies as well as the increase in the infant population over time in Burkina Faso. Epidemiologically, data on intussusception is lacking at the population level in most African countries. Such statistical data should provide a solid basis for effective control of this disease. The highest incidence in the world was reported by Tran et al [14] in Hanoi, Vietnam with 196 cases per 100,000 person-years for children under 5 years of age. A study by Kamdem et al in France showed an incidence of 0.48 per 1,000 live births [2].

Intussusception usually occurs in children under one-year-old with a median and a peak between 6 months and 8 months [2, 3] as was observed in our study. This age distribution may be due to factors associated with nutritional disorders related to weaning. At the etiological level, only 3.7% of the intussusception had an etiology in our series. Secondary intussusception is rare in children under 5 [2, 3, 14]. In our context following rotavirus vaccine introduction, monitoring the risk of intussusception following vaccination is important., although studies have shown that the risk of developing intussusception following rotavirus vaccine is negligible in low-income settings [9, 11].

The delay of consultation has a potential impact on treatment outcomes as other authors have found [1, 3]. While a higher proportion of children who died had delayed treatment compared with survivors, this difference was not statistically significant, possibly due to a low sample size. Association between intestinal resection and mortality was observed, probably due to higher rate of post-surgical complications. Since intestinal necrosis and parietal edema, the main factors of intestinal resection, are related to delayed management, consultation delay is a real morbidity-mortality factor in the IS. Ngendahayo et al also made the same observation [3].

The diagnosis of intussusception must be made early. The classic triad of symptoms which occurred in 24.3% of patients in this evaluation reflects an already advanced stage of the disease [2]. This delay was even more evident in 3.8% of patients who had prolapse at the anus. These findings highlight the need to promote early diagnosis through the continuous training of health personnel, and to increase the awareness of the population to recognize the main signs of intussusception in infants, that is paroxysmal abdominal pain with unexplained screaming and crying followed by a period of calm. Any suspicious case should trigger performance of an abdominal ultrasound. This exam was performed only in 75.5% of the patients in our series. Ultrasound diagnosis is rarely needed with late presentations, which have a clear clinical picture, an incorporated triad, with the palpation of a sausage-shaped lump on exam, or the presence of prolapse.

The therapeutic enema performed in our series under ultrasound control with tap water is an alternative to promote for developing countries because it is effective and of a lower cost [15]. Olumide in Nigeria and Mensah in Ghana practiced this method in their series with respectively 42.3% and 75% success rates [16, 17]. The use of enemas for treatment of intussusception is common in Western countries [2, 14]. In the series of Kamdem et al in France, the therapeutic enema was performed in 85.6% of patients [2]. It must take precedence over the surgical treatment that is still widely practiced in Africa [1, 3, 4, 11]. Once the abdominal surgical approach is performed, the risk of excessive intestinal resection is substantial [3, 4]. Bowel resection is a risk factor for mortality among intussusception cases. To avoid this, every effort should be made to diagnose intussusception early.

## Conclusion

Intussusception is a serious surgical pathology and treatment delays are common in our setting. Delayed presentation contributed to considerable morbidity and mortality. Training the health staff of the local hospitals and raising awareness of the people to consult early after the warning signs will help to enhance early diagnosis of intussusception. In addition, the accessibility of the ultrasound and the training of specialists in the fields relating to the pediatric pathology are also important. This information will contribute to the early diagnosis and non-operative treatment of intussusception and to the detection of other serious and frequent malformative pathologies. This study provides an update data on the epidemiology and management of intussusception in a hospital in Burkina Faso before the introduction of the rotavirus vaccine. This will serve as a benchmark for carrying out the surveillance of this pathology as a major adverse event following immunization with rotavirus vaccine.

### What is known about this topic

- Acute intestinal intussusception is the leading cause of acute intestinal obstruction in infants and young children;
- Data on the epidemiology and clinical presentation of intussusception is limited in Africa.
- Rotavirus vaccines have been associated with intussusception in some setting but not in sub-Saharan Africa.

### What this study adds

- This study provides an update data on the epidemiology and management of intussusception in a hospital in Burkina Faso before the introduction of the rotavirus vaccine;
- In Burkina Faso, delays in seeking care were common and were associated with mortality.
